# Targeting FLT3 Mutations in Acute Myeloid Leukemia

**DOI:** 10.3390/cells7010004

**Published:** 2018-01-08

**Authors:** Riad El Fakih, Walid Rasheed, Yousef Hawsawi, Maamoun Alsermani, Mona Hassanein

**Affiliations:** King Faisal Specialist Hospital and Research Center Riyadh, Riyadh 11211, Saudi Arabia; relfakih1@kfshrc.edu.sa (R.E.F.); wrasheed@kfshrc.edu.sa (W.R.); yhawsawi@kfshrc.edu.sa (Y.H.); MALSERMANI@kfshrc.edu.sa (M.A.)

**Keywords:** FMS-like tyrosine kinase 3, acute myeloid leukemia, Midostaurine

## Abstract

The FMS-like tyrosine kinase 3 (FLT3) pathway has an important role in cellular proliferation, survival, and differentiation. Acute myeloid leukemia (AML) patients with mutated FLT3 have a large disease burden at presentation and a dismal prognosis. A number of FLT3 inhibitors have been developed over the years. The first-generation inhibitors are largely non-specific, while the second-generation inhibitors are more specific and more potent. These inhibitors are used to treat patients with FLT3-mutated AML in virtually all disease settings including induction, consolidation, maintenance, relapse, and after hematopoietic cell transplantation (HCT). In this article, we will review the use of FLT3 inhibitors in AML.

## 1. Introduction

**Figure 1:**
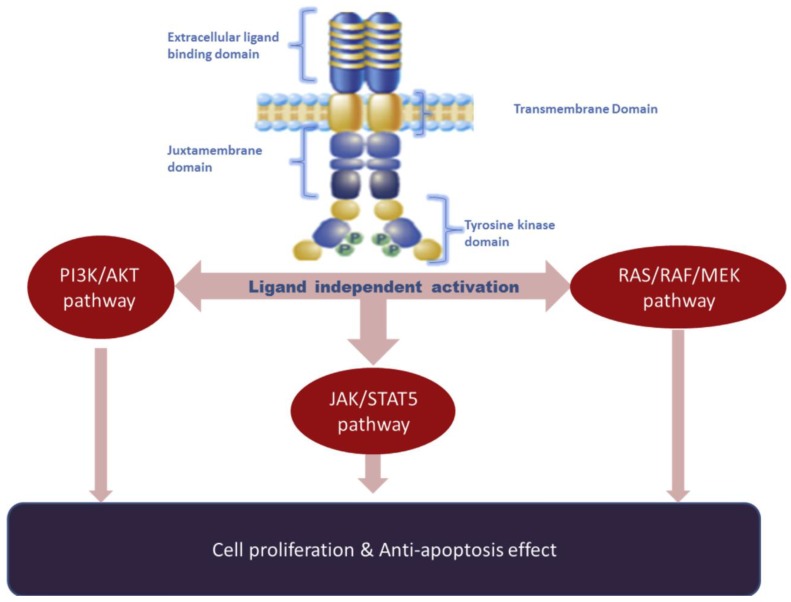
FLT3 activation pathway.

The FMS-like tyrosine kinase 3 (*FLT3*) gene, which is located on chromosome 13q12, encodes the FLT3 tyrosine kinase receptor expressed on the surface of CD34^+^ hematopoietic stem cells and other immature hematopoietic progenitors. It is a type-1 transmembrane receptor tyrosine kinase consisting of an extracellular domain, transmembrane domain, and an intracellular tyrosine kinase domain. FLT3 has five Ig domains in its extracellular domain and two kinase domains in its intracellular domain. FLT3 is a class III receptor tyrosine kinase, other members are platelet-derived growth factor receptor (PDGFR), macrophage colony-stimulating factor receptor (FMS), and stem cell factor receptor (c-KIT). Like other class III members, upon activation, the FLT3 tyrosine kinase promotes the activation of downstream pathways involving phosphatidylinositol-3 kinase (PI3K), AKT, mammalian target of rapamycin (mTOR), RAS, and extracellular signal-related kinase (ERK) (Figure 1). Although the activation of the receptor is normally ligand dependent, mutations also constitutivelly activate the receptor and the cells uncontrolled proliferation [1]. Acute myeloid leukemia (AML) is a heterogeneous disease characterized by multiple genetic aberrations [2]. Approximately 20–30% of AML patients carry an internal tandem duplication (ITD) mutation in the *FLT3* gene, which leads to uncontrolled cellular proliferation, survival, and differentiation through constitutive activation of FLT3 [3]. The prognostic impact of the ITD of FLT3 (FLT3-ITD) depends on the allelic ratio. Studies have shown that patients with a low FLT3-ITD allelic ratios (<0.5) have a favorable prognosis in the presence of a nucleophosmin (NPM1) mutation, in comparison to those without FLT3-ITD in the presence of NPM1 mutation. On the other hand, patients with a high FLT3-ITD allelic ratios (≥0.5) carry a dismal prognosis in the absence of an NPM1 mutation [4,5,6], and these are considered as one of the adverse risk groups in the 2017 European LeukemiaNet risk stratification [7].

Mutations in the tyrosine kinase domain (TKD) of FLT3 are less frequent (7%) and currently have no clinically significant impact [8]. FLT3-mutated AML is frequently found in patients with cytogenetically normal AML [9] and portends a poor prognosis in these patients [10], especially those less than 60 years old [11]. The Southwest Oncology Group (SWOG) trial 9031 enrolled 140 elderly AML patients aged over 55 years, demonstrated no significant impact of ITD mutations on the overall survival of patients with mutations (34%) [12]. Another study enrolled 380 AML patients, 12% of whom had an FLT3-ITD mutation, and also showed no impact of FLT3-ITD on the outcome of the elderly AML patients [13]. However, two retrospective studies showed poor outcomes for FLT3-mutated elderly patients [14,15]. A number of trials already published and multiple other trials are underway to investigate the effects of targeting FLT3 on the outcomes of AML patients.

## 2. Targeting FLT3 Mutations in AML

The prognostic impact of FLT3 mutations has made FLT3 an interesting target. In preclinical studies, FLT3 inhibitors were capable of inhibiting FLT3 phosphorylation and inducing apoptosis of the cell as a result [16,17].

In early clinical studies using non-selective FLT3 inhibitors such as sunitinib and lestaurtinib, which usually target more than one member of class III tyrosine kinases, however, high drug concentrations were needed to induce sustained inhibition. Recent development of more specific FLT3 inhibitors led to a more constant effect and better tolerability than those non-selective inhibitors (Table 1) [18]. Despite that, responses to FLT3 inhibitors are usually transient due to the emergence of resistant mutations [19]. The acquisition of point mutations in the ATP binding site of the TKD of FLT3 is the primary cause of resistance to two commonly used FLT3 inhibitors: midostaurin [20] and sorafenib [21]. Other proposed mechanisms of resistance include the stimulation of antiapoptotic proteins such as BCL2, MCL1, and BCL-x [22], and the activation of different pro-survival pathways, including MEK/ERK, PI3K/AKT/mTOR, and STAT5/PIM pathways, in addition to increased expression of FLT3 ligands [23].

## 3. Non-Selective FLT3 Inhibitors

### 3.1. Sunitinib (SU11248)

Sunitinib is a multitargeted tyrosine kinase inhibitor that can inhibit c-KIT, kinase insert domain receptor (KDR), and PDGFR kinases, as well as FLT3. Currently, it is used for treating renal cell carcinoma, gastrointestinal stromal tumors, and neuroendocrine tumors [24]. In AML with FLT3-ITD, sunitinib has led to short remissions and significant toxicities as a single agent in a phase I study [25]. In phase I/II trials recruited elderly AML patients with FLT3 mutations, sunitinib in combination with intensive chemotherapy demonstrated a complete remission/complete remission with incomplete hematological recovery (CR/CRi) rate of 59% with relapse-free survival of 1 year. Dose-limiting toxicity (DLT) included prolonged hematotoxicity and hand–foot syndrome [26].

### 3.2. Lestaurtinib (CEP701)

Lestaurtinib targeting both mutant and wild-type JAK2 is also a potent FLT3 inhibitor. It showed biological and clinical activity when used as a monotherapy in patients with relapsed/refractory AML [27]. In a subsequent phase II trial that included old, unfit, and previously untreated AML patients with either mutated or wild-type FLT3, lestaurtinib treatment as a single agent for 8 weeks led to non-sustained reduction in blasts [28]. In an intention to study salvage chemotherapy followed by lestaurtinib, 29 patients in the lestaurtinib arm achieved CR or partial remission (PR), while no overall survival (OS) rate difference was observed in comparison with that of 23 patients in the control arm. Of note, more patients in the lestaurtinib arm than those in the control arm discontinued the treatment due to adverse events [29]. In the UK MRC AML15 and the NCRI AML17 studies, lestaurtinib failed to demonstrate any significant clinical benefit in terms of remission and survival rates when added sequentially to standard front-line chemotherapy to treat FLT3-mutated AML patients included in those two prospective randomized clinical trials. However, correlative studies indicated improved OS and reduced rates of relapse in patients who achieved sustained FLT3 inhibition of >85% [30].

### 3.3. Tandutinib

Tandutinib was able to inhibit proliferation of hematopoietic stem cells in vivo, which have FLT3-ITD mutations as well as mutations in PDGFR or c-KIT [31]. In a clinical phase I study, tandutinib showed some activity for patients with refractory or relapsed AML with FLT3-ITD mutations. However, the DLT was mainly muscle weakness that was reversible, as well as manageable nausea and vomiting [32].

### 3.4. Quizartinib (AC220)

Quizartinib is a second-generation inhibitor against multiple kinases that was developed to treat FLT3-mutated AML; it is also active against AML with PDGFR or c-KIT mutations [33]. The dosing regimen of quizartinib was tested in a phase I study, which included relapsed/refractory AML patients with and without FLT3 mutations. In this study patients received quizartinib in two dose regimen either daily for a 28-day cycle or intermittently (2 weeks on and 2 weeks off); grade 3 QTc prolongation was the DLT [34]. Despite the promising response rates (61% to 72%) to quizartinib treatment as a single agent in phase II studies—which included patients with relapsed and refractory AML and even patients who had relapsed after hematopoietic stem cell transplantation (HCT)—relapse within the first 3 months occurred in up to 50% of the patients [35,36].

### 3.5. Sorafenib

Sorafenib is a multitargeted agent approved for the treatment of hepatocellular carcinoma as it inhibits RAF-1, and renal cell carcinoma as it inhibits vascular endothelial growth factor receptor (VEGFR), as well as PDGFR and c-KIT [37]. Sorafenib is also a potent FLT3 inhibitor that led to a marked reduction in bone marrow and peripheral blood blasts in all patients with FLT3 mutations who were involved in a phase I study using sorafenib as a single agent against refractory or relapsed AML, while there was no DLT after oral administration with 200 to 400 mg twice daily [38]. Due to the safety profile, combined chemotherapy with other drugs including hypomethylating agents has further been conducted. In the relapse and refractory settings, sorafenib combined with idarubicin or high-dose cytarabine was safe, and induced overall CR rates from 75% to 92% of patients with mutated FLT3 [39]. Another phase II study in the relapse and refractory settings, which use sorafenib in combination with azacitidine, demonstrated an overall response rate of 46% with minimal toxicity. This allowed patients to have subsequently HCT [40]. Furthermore, a phase II study was carried out for combination therapy using sorafenib with idarubicin or cytarabine during induction and consolidation, followed by treatment with sorafenib as a single agent for up to 1 year as maintenance for patients who completed consolidation. The results showed very high CR rates with complete eradication of FLT3-mutated clones in more than 50% of the patients. After a median follow-up of 9 months (range, 1–16 months), however, 55% of patients relapsed [41]. Moreover, the randomized placebo-controlled SAL–SORAML trial that involved 267 younger patients with newly diagnosed AML reported better event-free survival and relapse-free survival but not OS in patients who received sorafenib vs. placebo in the upfront setting [42].

## 4. Selective FLT3 Inhibitors

### 4.1. Midostaurin (PKC412)

Midostaurin is active against both FLT3-ITD and FLT3-TKD mutations. When used as a single agent at a dose of 75 mg, 3 times daily, a significant reduction in peripheral blasts was seen in 70% of 20 patients studied. The treatment was well tolerated by the patients, aside from two fatal pulmonary events with unknown etiology [43]. The addition of midostaurin to induction and consolidation therapy in patients with newly diagnosed AML at a dose of 50 mg twice daily resulted in an 80% CR rate; the OS rate at 1 and 2 years was similar irrespective of FLT3 mutation status [44]. In a phase II randomized study, 95 patients with AML or high-risk myelodysplastic syndrome (MDS) were treated with oral administration of midostaurin at 50 or 100 mg twice daily. This treatment led to more than 50% reduction in circulating and bone marrow blasts and the response was more prominent in patients with FLT3 mutations (71% vs. 42%) [45]. Subsequently, a phase I trial studied the combination of midostaurin with all-trans retinoic acid and CLAG (cladribine, cytarabine, granulocyte colony-stimulating factor) chemotherapy for the treatment of relapsed/refractory AML and showed an overall response rate (ORR) of 33% (CR and CRi) [46]. The RATIFY study randomized over 700 young patients (range 18–59 years) with untreated AML and FLT3-ITD (77%) or FLT3-TKD (23%) mutation to receive midostaurin or placebo with induction chemotherapy. This multi-institutional, multinational, double-blind, randomized trial reported a significant improvement in OS of 23% (hazard ratio 0.77) in favor of midostaurin even when patients who underwent transplantation (57%) were censored; the benefit of midostaurin was consistent across FLT3 mutation type (ITD vs. TKD) and allelic mutation fraction (low vs. high). In addition, the event-free survival post transplantation was significantly improved in patients who were treated with midostaurin (hazard ratio 0.84; *p* = 0.025) [47,48]. Midostaurin is the first targeted therapy approved by the Food and Drug Administration for the treatment of FLT3-mutant AML in the US [49].

### 4.2. Gilteritinib (ASP2215)

Gilteritinib is a second-generation selective potent inhibitor of FLT3 and AXL (a member of the TAM receptor tyrosine kinase family). Results of a phase I/II trial of gilteritinib use in FLT3-mutated refractory/relapsed AML showed an ORR of 57% that reached 63% with higher drug doses (≥80 mg) [50]. Gilteritinib is now being tested in multiple phase III trials in comparison to other salvage regimens in the relapse/refractory setting (NCT02421939, NCT03182244), as maintenance in first CR following induction/consolidation (NCT02927262) or after allogeneic HCT (NCT02997202), and in combination with azacitidine vs. azacitidine alone in patients with FLT3-ITD ineligible for intensive chemotherapy (NCT02752035).

## 5. Future Directions

The main concern when using FLT3 inhibitors is the development of resistance. Several trials using different agents (e.g., AMG 925, SAR302503, ponatinib, G-749) were conducted to overcome this problem [51,52,53,54].

Crenolanib is a pan-selective FLT3 inhibitor believed to bypass resistance caused by the development of TKD mutations in the activation loop, which is the main mechanism of resistance to quizartinib [55]. However, in a phase II study, crenolanib showed better activity in FLT3 inhibitor-naive patients compared with previously treated patients [56], and currently it is being tested in the front-line setting in a phase III trial comparing crenolanib vs. midostaurin after induction and consolidation (NCT03258931). Another phase III trial is comparing chemotherapy combined with crenolanib vs. chemotherapy alone in the relapse/refractory setting in patients with mutated FLT3-ITD AML (NCT02298166).

Targeting other survival pathways together with FLT3 is an interesting approach. Multiple in vitro experiments showed synergism by combining midostaurin with an mTOR or PI3K inhibitor and sunitinib with an mTOR or MAPK–ERK1/2 inhibitor [57,58,59,60]. A phase I trial combining the mTOR inhibitor RAD001 with midostaurin is ongoing (NCT00819546).

Patients with FLT3-ITD AML have a high rate of relapse even after allogeneic HCT. FLT3 inhibitors used as post-transplant maintenance to reduce the risk of relapse is being actively investigated. Quizartinib treatment as a maintenance therapy for FLT3-mutated AML patients in CR post transplantation was investigated in a multicenter phase I study. Only one out of 13 patients experienced relapse in that study (despite using doses <60 mg) [61]. Another phase I study of sorafenib maintenance post transplantation starting between day 45 and 120 showed that the drug is well tolerated and the OS was impressive at 95% at one year post HCT [62]. These highly promising results deserve further evaluation in randomized clinical trials.

## Figures and Tables

**Table 1:** Phases of development and major toxicities of FMS-like tyrosine kinase 3 (FLT3) inhibitors.

FLT3 Inhibitors	Selectivity	Targets	Phases of Development	Toxicity
Sunitinib (SU11248)	Non-selective	c-KIT, KDR PDGFR, and FLT3	Phase II [26]	Decreased appetite, headache, GI symptoms
Lestaurtinib (CEP701)	Non-selective	Mutant and wild-type JAK2, and FLT3	Phase II [28] Phase III [29,30]	Infections, sepsis, myocardial infarction
Tandutinib (CT53518)	Non-selective	PDGFR, c-KIT, and FLT3	Phase I [32]	Muscle weakness
Quizartinib (AC220)	Non-selective	Multikinase inhibitor PDGFR, c-KIT, and FLT3	Phase I [34] Phase II [35,36]	QTc prolongation
Sorafenib	Non-selective	RAF-1, VEGFR, PDGFR, c-KIT, and FLT3	Phase I [38,39] Phase II [39,40,41] Phase III [42]	Skin rash, fatigue, diarrhea
Midostaurin (PKC412)	Selective	FLT3-ITD and FLT3-TKD	Phase I [43,44,46] Phase II [45] Phase III [47,48]	Fever, flu-like symptoms, mouth sores, unusual bleeding or bruising
Gilteritinib (ASP2215)	Selective	FLT3/AXL	Phase I/II [50] Phase III [ongoing]	Diarrhea, fatigue, high liver function tests (LFT)

c-KIT, v-kit Hardy–Zuckerman 4 feline sarcoma viral oncogene homolog; KDR, kinase insert domain receptor; PDGFR, platelet-derived growth factor receptor; FLT3, FMS-like tyrosine kinase 3; JAK2, Janus Kinase 2; VEGFR, vascular endothelial growth factor receptor; RAF-1, v-raf-1 murine leukemia viral oncogene homolog 1; AXL, a member of the TAM receptor tyrosine kinase family.
